# Cost-effectiveness analysis of first-line tislelizumab plus chemotherapy for extensive-stage small cell lung cancer from the perspective of the healthcare system in China

**DOI:** 10.3389/fpubh.2025.1552734

**Published:** 2025-05-30

**Authors:** Feng Chen, Xue Feng, Lin Lin Xiao, Hai Juan Tang, Shu Xia Qin, Jie Ping Peng, Jing Bai

**Affiliations:** ^1^Department of Respiratory and Critical Care Medicine, The First Affiliated Hospital of Guangxi Medical University, Nanning, Guangxi, China; ^2^Department of Health Economics, School of Information and Management, Guangxi Medical University, Nanning, Guangxi, China; ^3^Department of Pharmacy, The First Affiliated Hospital of Guangxi Medical University, Nanning, Guangxi, China

**Keywords:** small-cell lung cancer, immune checkpoint inhibitors, cost-effectiveness analysis, tislelizumab, first-line therapy

## Abstract

**Background:**

Extensive-stage small-cell lung cancer (ES-SCLC) poses a formidable challenge due to its aggressive nature and poor prognosis. While immune checkpoint inhibitors have shown promise as part of first-line therapy, their cost-effectiveness and survival benefits in the Chinese healthcare system are not well understood. This study evaluates the cost-effectiveness of first-line tislelizumab combined with chemotherapy versus chemotherapy alone for ES-SCLC.

**Methods:**

We conducted a cost-effectiveness analysis using a partitioned survival mode (PSM) to compare tislelizumab plus chemotherapy versus chemotherapy alone for the first-line treatment of ES-SCLC. The model integrated survival estimates from the RATIONALE-312 Phase III clinical trial, direct medical costs, and quality-adjusted life year (QALY) sourced from the literature. We calculated 10-year cost per QALY gained from Chinese healthcare system perspective. The analysis of cost-effectiveness was benchmarked against a willingness-to-pay threshold three times of GDP per capita in China. Sensitivity analyses were conducted to evaluate parametric uncertainty and model robustness.

**Results:**

Compared to the chemotherapy alone group, the tislelizumab plus chemotherapy group resulted in an incremental cost-effectiveness ratio (ICER) of US$31,072.79 per quality-adjusted life-year (QALY), which is below the threshold of US$37,765 per QALY. Sensitivity analyses indicated that the utility value of progression-free survival (PFS) is a principal determinant of the ICER, with the ratio fluctuating between $27,246 and $36,417 per QALY, well below the willingness-to-pay threshold. In scenario analyses, tislelizumab plus chemotherapy resulted in an ICER of US$ 38,665.59/QALY with PET-CT imaging (exceeding the $37,765/QALY threshold) but was cost-effective at US$ 30,076.37/QALY when imported topotecan was used as second-line treatment.

**Conclusion:**

Tislelizumab plus chemotherapy demonstrates cost-effectiveness in the first-line treatment of ES-SCLC in China. This study provides preliminary evidence for the economic value of tislelizumab in the treatment of ES-SCLC, supporting its consideration as a first-line therapeutic option.

## 1 Introduction

Lung cancer persists as the predominant contributor to global cancer incidence and mortality, with 2.5 million new cases diagnosed in 2022 (12.4% of total malignancies) (1). Epidemiological surveillance in China demonstrates age-standardized incidence (ASIR) and mortality rates (ASMR) of 40.8 and 26.7 per 100,000 population, respectively, exceeding all other cancers. Notably, lung cancer displaced gastric cancer as the leading driver of disability-adjusted life years (DALYs) in 2021, reversing the 1990 disease burden hierarchy (2). This transition has precipitated sustained economic strain on public health systems (3).

Small cell lung cancer (SCLC), a neuroendocrine tumor, accounts for approximately 15% of all lung cancers (4). This aggressive cancer is often diagnosed at an advanced stage, with around two-thirds of patients presenting with distant metastases (5). While chemotherapy typically yields favorable initial responses, many patients experience relapse and develop resistance to treatment (6, 7). SCLC also presents with limited actionable gene mutations and lacks effective targeted therapies, resulting in poor long-term outcomes and therapeutic challenges (8). Historically, chemotherapy and radiotherapy have been the standard treatments for ES-SCLC (6, 9). Despite ongoing research into anti-angiogenic therapy, there have been no breakthroughs in first-line treatment (10). A major breakthrough occurred in 2019 with the IMpower133 clinical trial, which demonstrated that combining atezolizumab, an immune checkpoint inhibitor, with chemotherapy significantly improved patient outcomes compared to chemotherapy alone in patients with ES-SCLC (11). This finding marked a pivotal shift in treatment after two decades of limited progress. Subsequently, clinical trials such as CASPIAN and ASTRUM-005 have yielded favorable outcomes with regard to immune checkpoint inhibitors in patients with ES-SCLC (12, 13). In addition, immune checkpoint inhibitors such as durvalumab, serplulimab and adebrelimab have been granted approval for the first-line treatment of ES-SCLC (12–14).

Tislelizumab is a well-utilised PD-1 inhibitor that has been extensively employed in an array of tumor therapies. This is due to the high affinity of its Fab segment for the PD-1 receptor and the diminished ADCP effect of the modified Fc segment, which effectively mitigates T-cell depletion and exhibits a prolonged half-life (15, 16). The results of the RATIONALE-312 Phase III trial, published in May 2024, demonstrated that tislelizumab combined with chemotherapy significantly improved overall survival (OS:HR = 0.75 [95% CI: 0.61–0.93]; *p* = 0.0040) and progression-free survival (PFS: HR = 0.64 [95% CI: 0.52–0.78]; *p* < 0.0001) compared to chemotherapy alone (17). In light of the aforementioned data, the application for tislelizumab as a first-line treatment for patients with ES-SCLC was accepted by the National Drug Administration (NMPA) in June 2024 and recommended in the Chinese Society of Clinical Oncology (CSCO) guideline as Class 1A, Level 3 evidence (18).

The existing body of research regarding the cost-effectiveness of tislelizumab in conjunction with chemotherapy versus chemotherapy alone is underdeveloped. The study conducted by Lang et al. (19) did not comprehensively reflect the clinical realities specific to the Chinese healthcare context. This dearth of economic data hinders a comprehensive appreciation of the economic implications of tislelizumab in the treatment of advanced extensive-stage small cell lung cancer. Consequently, the objective of this study was to evaluate the incremental cost-effectiveness ratio (ICER) of tislelizumab combined with chemotherapy in comparison to chemotherapy alone, from the vantage point of the Chinese healthcare system. The findings of this study are anticipated to inform the optimal allocation of healthcare resources, balancing the financial sustainability and clinical efficacy of the treatment protocol.

## 2 Methods

### 2.1 Patient population and intervention

The patient characteristics and interventions in this study were based on the RATIONALE-312 trial, a phase III, multicentre, randomized, double-blind, placebo-controlled clinical trial conducted in China (17). Since there was no direct involvement of human subjects, no institutional review board (IRB) approval or ethics committee waiver was required. Eligible patients were over 18 years old, with histologically or cytologically confirmed extensive SCLC, as per the diagnostic criteria defined in the seventh edition of American Joint Committee on Cancer (AJCC). Patients were required to have an ECOG score of less than 1, a life expectancy of at least 12 weeks, and no prior systemic treatment for extensive SCLC. The study included 457 patients, randomized in a double-blind manner (1:1) to receive either tislelizumab or a placebo (17). Both groups received four cycles of induction chemotherapy with a regimen of etoposide (100 mg/m^2^, intravenously on days 1–3 of every 21 days) in combination with a platinum-based agent (cisplatin 75 mg/m^2^ or carboplatin, with the dose calculated as the plasma or serum area under the concentration-time curve equal to 5) in combination with 200 mg of tislelizumab or placebo on the first day of each cycle (17). After cycles of induction chemotherapy, the tislelizumab group continued to receive maintenance therapy, while the placebo group continued to receive placebo until disease progression, loss of clinical benefit, occurrence of unacceptable toxicity or patient withdrawal of consent, for 21 days per treatment cycle (17). Patients underwent an assessment with an imaging review every 2 cycles. The demographic and baseline characteristics were found to be well-balanced across the two treatment groups (17).

### 2.2 Model structure

**Figure 1:**
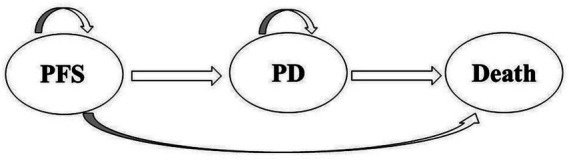
Model structure. PFS, progression-free survival; PD, progressive disease.

The analysis utilized TreeAge Pro 2022 software (Williamstown, MA, USA) and R software (version 4.2.3, Vienna, Austria) to structure a partitioned survival model, an approach for evaluating the economic impact of tislelizumab plus chemotherapy versus chemotherapy alone. This model simulates three distinct health states in patients with extensive small cell lung cancer (SCLC): progression-free survival (PFS), progressive disease (PD), and death (20–22) (Figure 1). The model estimates the proportion of individuals in each health state by employing a series of independently simulated, non-mutually exclusive survival curves. As a result, it is widely used in cost-effectiveness analyses for cancer treatments (23). As the model indicated a 99% mortality rate over 10 years, the time horizon chosen in this study was 10 years. A cycle length of 3 weeks was established to align with the chemotherapy regimen of the RATIONALE-312 trial (17). we conducted the study from the Chinese healthcare system’s perspective. The primary output of the model is the incremental cost-effectiveness ratio (ICER), which assesses treatment cost-effectiveness against a specified willingness-to-pay (WTP) threshold. The analysis focused on direct healthcare costs from the perspective of the Chinese healthcare system, in accordance with China’s 2020 Guiding Principles for Pharmacoeconomic Evaluation (24). The cost-effectiveness analysis utilized a WTP threshold of 3 times China’s per capita GDP (37,765 US$/QALY, obtained in 2023) (24). All costs were converted to US dollars at an exchange rate of 1 USD to 7.0985 RMB (Last updated 4 November 2024).

### 2.3 Survival estimates and model transitions

To derive OS and PFS survival curves from RATIONALE-312, we used WebPlotDigitizer to extract lifetime data. R language software (version 4.2.3) was employed to reconstruct individual event occurrence times and extrapolate the survival curves. Various parametric survival models including exponential, Weibull, log-normal, log-logistic, Gompertz, and generalized gamma were utilized for curve fitting. The goodness of fit for each model was assessed through visual inspection and statistical criteria (Akaike Information Criterion and Bayesian Information Criterion) to identify the best-fitting model. Lower values of AIC and BIC indicated better fit (25). Results from the OS and PFS models in the tirilizumab + chemotherapy group and PFS in the chemotherapy group were distributed using a log-logistic distribution, and due to the presence of curve overlap, OS curves in the chemotherapy group were distributed using a log-normal distribution. Key Parameters of the best-fitted distributionsre were presented in Table 1.

**Table 1:** Parameters of the best-fitted distributions.

Kaplan meier survival curve	Best fitted distribution	Key parameters
OS curve of tislelizumab plus chemotherapy	Log-logistic	shape (*γ*) = 0.57, scale (*λ*) = 0.31
PFS curve of tislelizumab plus chemotherapy	Log-logistic	shape (γ) = 0.64, scale (λ) = −0.75
OS curve of Placebob plus chemotherapy	log-normal	μ = 0.14, σ = −0.27
PFS curve of Placebob plus chemotherapy	Log-logistic	shape (γ) = 1.38, scale (λ) = −0.94

OS, overall survival; PFS, Progression-Free Survival; AEs, adverse events.

### 2.4 Cost input

This analysis considered only direct medical costs, including medication, intravenous administration, routine laboratory tests, tumor imaging, best supportive care, terminal care, and expenses related to severe adverse events (AEs). All cost parameters are listed in Table 2. As SCLC patients do not usually undergo driver gene testing, and in consultation with experienced clinical experts, we assumde that the costs of routine investigations, imaging, intravenous injections and optimal supportive care were the same for both treatment options. The terminal care costs represented the resources used when patients were dying. In this study, we used conventional CT combined with MRI as a means of imaging assessment. In the base case analysis, we used an average patient age of 62 years, an average weight of 65 kg, and a body surface area (BSA) of 1.72 m^2^ for drug dosage calculations (17, 26). In the RATIONALE-312 study, the experimental group was treated with 200 mg of tislelizumab, and 55 and 67% of patients in the tislelizumab and placebo groups, respectively, received second-line treatment (17). Following the Chinese Society of Clinical Oncology (CSCO) guidelines (18), we assumed that the second-line regimen would be single-agent topotecan (1.2 mg/m^2^ given intravenously on days 1–5 every 21 days). Patients underwent 1 imaging assessment every 2 cycles. Due to the significant uncertainty of third-line treatment, we assumed that patients not receiving second-line treatment received best supportive treament. Only grade 3 or higher AES with an incidence greater than 5% were included in this study because grade 1–2 AES can usually be effectively managed (assuming AES occur only in the first cycle of PD or PFS status). Incidence data of adverse events (AEs) were sourced from clinical trials (17). Cost for all drugs were obtained from the China Data Platform^1^ and additional direct medical costs were obtained from previous studies (27, 28). Costs and QALYs were calculated using a discount rate of 5% according to the China Guidelines for Pharmacoeconomic Evaluations 2020 (24). The cost of PET-CT obtain from the Local hospital data.

**Table 2:** Key model inputs.

Parameters	Baseline value	range	Reference	Distribution
Drug cost, US$/per cycle				
Tislelizumab	357	286–428	National databases	Gamma
Platinum	66	53–79	National databases	Gamma
Etoposide	7	6—9	National databases	Gamma
Imported topotecan	572	458–686	National databases	Gamma
Homemade topotecan	72	58–86	National databases	Gamma
Cost of terminal care per patient	2,221	1777–2,665	(29)	Gamma
Cost of administration per cycle	36	29–43	(29)	Gamma
Cost of laboratory per cycle	166	133–199	(29)	Gamma
Cost of PET-CT per cycle	1,035	828–1,242	Local data	Gamma
Cost of tumor imaging per cycle	507	406–608	(29)	Gamma
Cost of best supportive treatment per cycle	221	177–265	(29)	Gamma
Cost of AEs, $				
Tisle +chemo	386	309–463	(27, 29)	Gamma
Placebo +chemo	309	247–371	(27, 29)	Gamma
Utilities				
Utility PFS	0.673	0.538–0.808	(26, 27)	Beta
Utility PD	0.473	0.378–0.568	(26, 27)	Beta
Disutilities				
Disutility for main AEs in chemo	0.142	0.113–0.17	(28, 29)	Beta
Disutility for main AEs in tislel plus chemo	0.126	0.11–0.15	(28, 29)	Beta
Risk for main AEs				
Risk for main AEs in chemo	0.79	0.632–0.948	(17)	Beta
Risk for main AEs in Tislel plus chemo	0.73	0.584–0.876	(17)	Beta
Rate of subsequent anticancer therapy				
chemo group	0.67	0.536–0.8	(17)	Beta
Tislel plus chemo group	0.55	0.44–0.66	(17)	Beta
Other model input				
Weight (Kg)	65	52–78	(17, 26)	Normal
Body surface area (meters2)	1.72	1.38–2.06	(17, 26)	Normal
Area under the curve (mg/mL/min)	5	NA	(17, 26)	Uniform
Serum creatinine (mg/dL)	1	NA	(17, 26)	Uniform
Discount rate	5%	1–8%	(24)	Beta

AEs, adverse events; Chemo, chemotherapy; OS, overall survival; PFS, Progression-Free Survival; Tislel, Tislelizumab.

### 2.5 Utility estimates

Health utility values, anchored on a 0 (death) to 1 (perfect health) scale, quantified patient quality of life (QoL) across disease states. Utilities for progression-free survival (PFS) and progressive disease (PD) health states (0.673 vs. 0.473, respectively) and disutility adjustments for severe adverse events (AEs) were systematically derived from published cost-effectiveness studies (27–29) (Table 2). The disutilities for AEs in both groups were calculated as the weighted sum of the disutilities for each AE and the Risk of the corresponding AEs (Supplementary Table 1). Incidence data for AEs were sourced from clinical trials (17).

### 2.6 Sensitivity analyses

One-way sensitivity analyses was applied to assess the impact of individual parameters on the ICER. Critical parameters included, drug cost, cost of AEs, utilities, disutilities, risk for main AEs. Per the China Guidelines for Pharmacoeconomic Evaluations (24), parameters with undefined variation ranges were assigned ±10–20% thresholds around baseline means, while a ± 20% interval informed by prior cost-effectiveness analyses was applied to each parameters (Table 2). The results of the one-way sensitivity analyses were presented by tornado plot, which are a valuable graphical tool to illustrate the comparative importance of individual parameters on the ICER. In the probabilistic sensitivity analyses (PSA), parameter distributions were determined according to established guidelines for health economic evaluation. For example, a gamma distribution was used for the cost parameters and a beta distribution for the AE incidence and utility parameters (30). A total of 10,000 Monte Carlo simulations were performed for the PSA. The results of the PSA were visualized using cost-effectiveness acceptability curves and a scatter plot.

### 2.7 Scenario analyses

In the present investigation, two distinct scenario analyses were conducted to evaluate their potential influence on the incremental cost-effectiveness ratio (ICER) outcomes. The first scenario involved the utilization of PET-CT as a means of imaging assessment, with patients undergoing assessments at every 2 cycles. In the second scenario, it was hypothesized that patients would receive imported topotecan as their second-line therapeutic regimen. Due to the superior diagnostic efficacy of PET-CT in small cell lung cancer (SCLC), it can significantly enhance staging and treatment planning (31). Moreover, the costs associated with PET-CT and imported topotecan are considerably higher compared to conventional diagnostic and therapeutic methods. Therefore, we conducted scenario analyses to assess the impact of these factors on cost-effectiveness.

## 3 Result

### 3.1 Base-case analysis

From the perspective of the chinese health care system, the total cost of tislelizumab plus chemotherapy was US$ 23,903 and US$ 15,451.82 for the placebo combination chemotherapy. Compared with chemotherapy alone group, tislelizumab plus chemotherapy group has an increase in QALYs of 0.27. It estimated that the ICER was US$ 31,072.79 per QALY for ES-SCLC (Table 3). The ICER did not exceed our identified threshold of US$ 37,765/QALY.

**Table 3:** The results of cost-effectiveness.

Group	Cost (US$)	QALYs	Incremental cost (US$)	Incremental QALY	ICER (US$/QALY)
Tisle+chemo	23,902	1.01	8450.91	0.27	31072.79
Placebo+chemo	15,451	0.74	NA	NA	NA

Chemo, chemotherapy; ICER, increment cost-effectiveness ratio; QALY, quality-adjusted life-years; Tislel, Tislelizumab.

### 3.2 Sensitivity analysis

**Figure 2:**
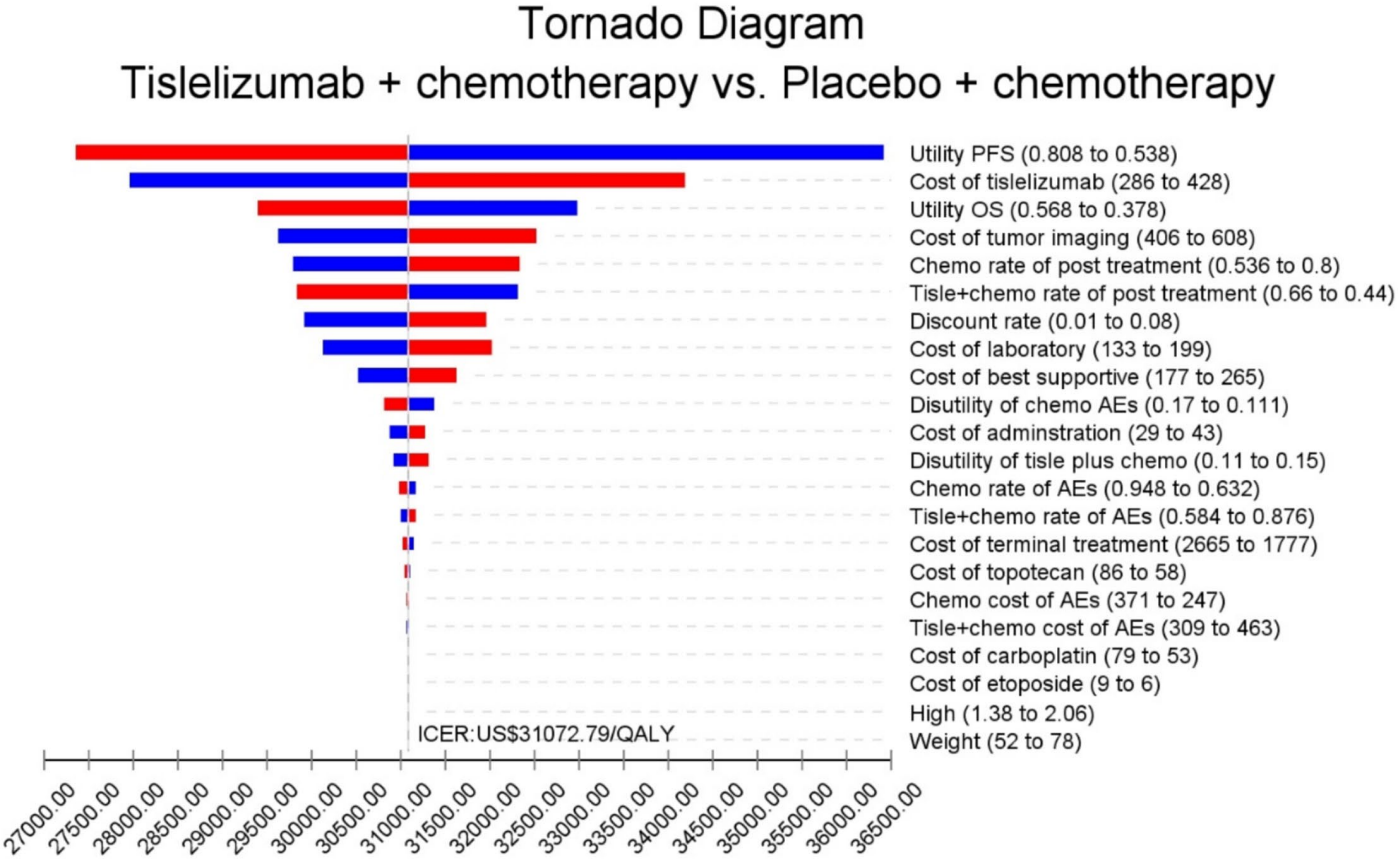
The outcomes of sensitivity analysis. AEs, adverse events; Chemo, chemotherapy; ICER, increment cost-effectiveness ratio; OS, overall survival; QALY, quality-adjusted life-years. PFS, progression-free Survival; Tislel, Tislelizumab; The parameter increases and the ICER is shown in blue. The parameter decreases and the ICER is shown in red.

**Figure 3:**
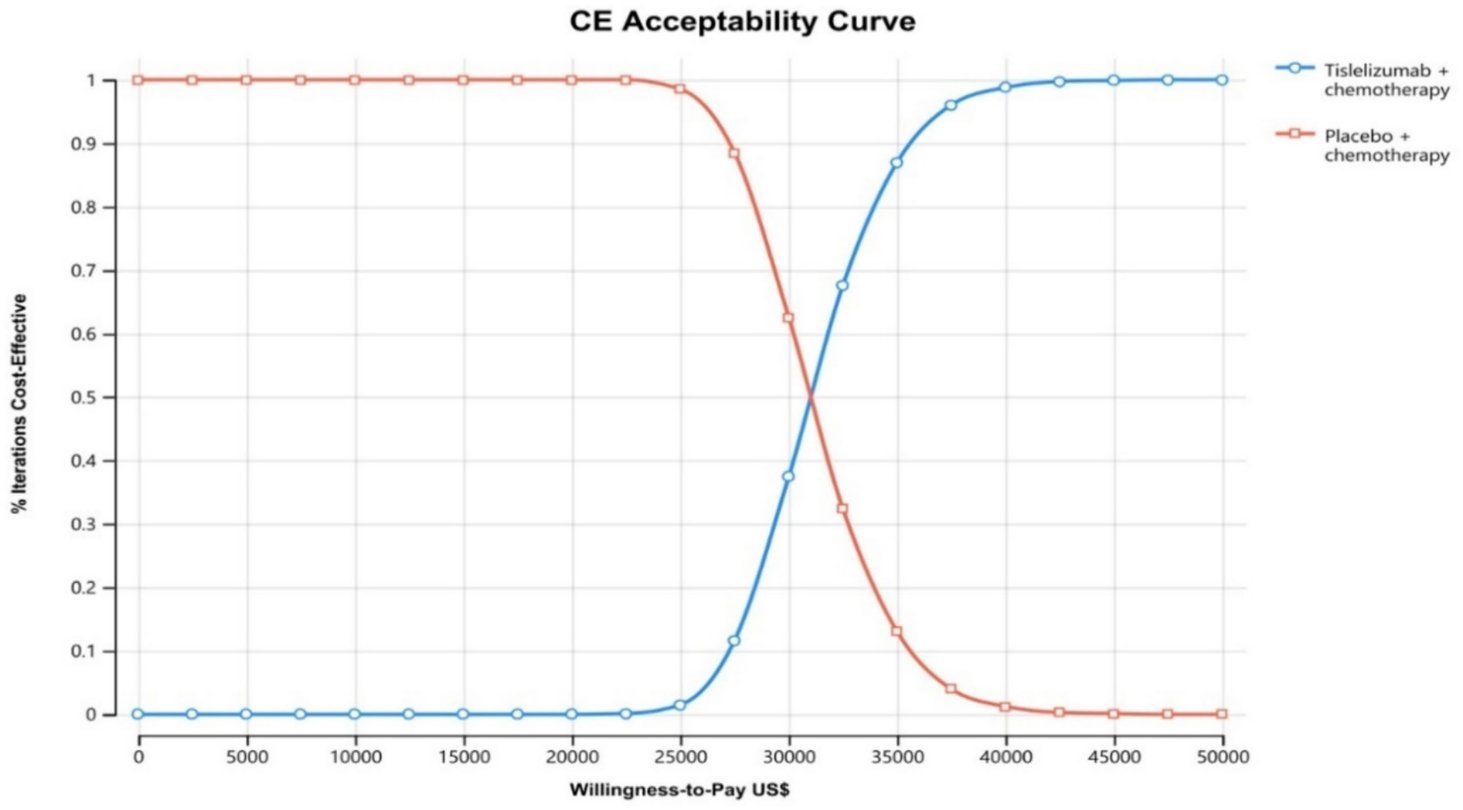
The cost-effectiveness acceptability curves for tirilizumab plus chemotherapy group compared to chemotherapy group. CE, Cost-effectiveness.

**Figure 4:**
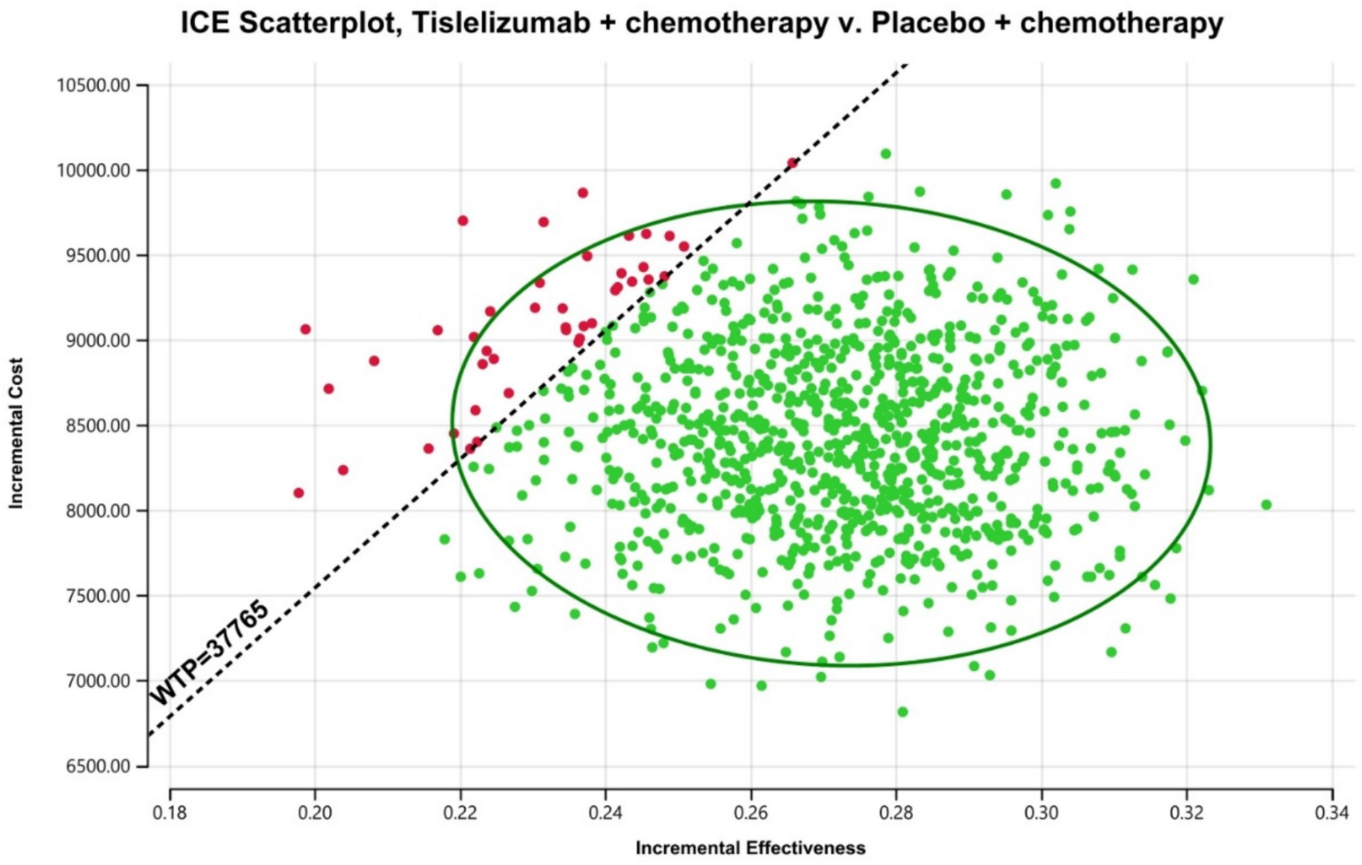
The outcomes of the ICER plane scatter plots. WTP, willingness-to-pay = 37,765 US$/QALY; WTP was the 3 times GPD per capita in China (2023).

The results of the one-way sensitivity analysis are shown in the tornado diagram (Figure 2). The utinity of PFS had the greatest impact on the ICER results. Variying the utilities by ±20% (to 0.538–0.808) moulded the incremental cost per QALY from US$ 27,246 and US$ 36,417 in patients with ES-SCLC. Despite adjusting the parameter variables, ICER did not exceed the set threshold of 37,765 US$ /QALY, demonstrating the reliability of our conclusions. The cost-effectiveness acceptability curves from the probabilistic sensitivity analyses show that the probability that the tislelizumab combination chemotherapy is cost-effective increases as the willingness-to-pay (WTP) threshold increases (Figure 3). Specifically, when the cost-effective benchmarks was set at US$ 37,765/QALY, the probability that the tislelizumab combination regimen was cost-effective compared to placebo combination chemotherapy in the overall population was 96% (Figure 4).

### 3.3 Scenario analyses

In the initial scenario analysis, employing PET-CT for patient imaging led to higher total costs for the plus chemotherapy group (US$ 32,466.87)versus the chemotherapy-only group (US$ 21,950.94). The tislelizumab plus chemotherapy improved effectiveness by 0.27 QALYs and increased costs by US$ 10,516, yielding an ICER of US$ 38,665.59/QALY for ES-SCLC, surpassing our threshold of US$ 37,765/QALY (Supplementary Table 3). In the second scenario, using imported topotecan as second-line treatment, the tislelizumab plus chemotherapy group still incurred higher total costs (US$ 29,537.77) compared to the chemotherapy alone group (US$ 21,357.86), with an ICER of US$ 30,076.37/QALY, which did not exceed our threshold of US$ 37,765/QALY (Supplementary Table 4).

## 4 Discussion

In the first-line treatment of extensive-stage small cell lung cancer (ES-SCLC), there are significant differences in the economic burden and survival benefit of different immune checkpoint inhibitor treatment regimens. Using an economic evaluation methodology, this study quantified the costs and effects associated with specific treatment regimens, providing a scientific basis for the National Healthcare Security Administration (NHSA) to determine coverage and reimbursement policies for innovative drugs. And this study was based on the RATIONALE-312 study conducted at 62 clinical trial sites in China (17), which reduced population selection and genetic bias and improved the reliability of the results.

A recent study comparing the cost-effectiveness of multiple immune checkpoint inhibitors in combination with chemotherapy versus chemotherapy alone in ES-SCLC showed that the incremental cost-effectiveness ratios (ICERs) of adebrelimab + chemotherapy, serplulimab + chemotherapy, atezolizumab + chemotherapy, durvalumab + chemotherapy, durvalumab + tremelimumab + chemotherapy were range from 80425.31 US$ to 270108.50 US$/QALY, compared to chemotherapy (32). Using three times China’s GDP per capita as the willingness-to-pay threshold, none of these immune checkpoint inhibitors were cost-effective (32). The results of Yi L et al. study (2024) for adebrelimab, atezolizumab and durvalumab were generally consistent with the results of previous cost-effectiveness analysis (CEA) studies (30, 33, 34). However, cost-effectiveness analyses of serplulimab are controversial across trials, possibly because of the wide variation in the cost of serplulimab used in different trials (26). Some studies may have considered scenario analysis of drug gifting. In addition, in a phase III clinical trial, serplulimab was used in a cross-line strategy in the first-line treatment of ES-SCLC, whereas the other drugs were only used in the first-line setting (33). This difference, if not taken into account, could have changed the final ICER results.

Lang et al. (19) study have assessed the cost-effectiveness of tislelizumab combined with chemotherapy for ES-SCLC. These analyses demonstrated an incremental cost-effectiveness ratio (ICER) of US$46,132.33 per QALY (19). This figure exceeds the cost-effectiveness threshold three times GDP per capita from a Chinese healthcare perspective (19). Our study introduces several methodological distinctions compared to precedent research, which may yield divergent incremental cost-effectiveness ratios (ICERs). Primarily, This study utilized a partitioned survival model (PSA), offering a distinct advantage over state transition models (STMs) by directly deriving patient proportions across health states from survival curves (35). The PSA’s robustness in reconstructing complex risk functions via extrapolation, without significant influence from the underlying survival model’s specifications, eliminates the need for additional assumptions on state transition probabilities, thus more accurately reflecting patient survival realities (36). This model is particularly adept for evaluating patients undergoing a sequence of non-reversible health states, aligning well with the economic assessment of antineoplastic drugs (37). Secondly, This study opted for conventional CT and MRI for imaging assessments, contrasting with the PET-CT employed in prior studies. While PET-CT excels in localizing metabolically active regions (38), it is more expensive and less sensitive in detecting brain metastases—a common occurrence in SCLC (39). To address PET-CT’s limitations, brain MRI is employed as a complementary diagnostic (39). Acknowledging the scarcity of PET-CT in Chinese hospitals and adhering to the Chinese COSCO guidelines, which endorse routine CT and MRI as level I, class 2A evidence (18), our approach is more pragmatic and applicable in clinical practice. Beyond the core analysis, scenario analyses within this study demonstrated that employing PET-CT for imaging assessments yielded an ICER of US$38,665.59/QALY for tislelizumab plus chemotherapy in ES-SCLC, exceeding three times GDP per capita threshold when compared to chemotherapy alone. This result suggested tislelizumab plus chemotherapy yield a high ICER. This outcome is likely due to the extended overall survival (OS) in the experimental group, which increased the number of necessary scans compared to the chemotherapy group, underscoring the significant role of imaging assessments in cost-effectiveness. Given the substantial differences in assumptions about second-line treatment regimens and drug pricing from previous studies, this study utilized the cost of domestically available generic topotecan as the second-line treatment drug, following CSCO guidelines (18). A subsequent scenario analysis, assuming the utilization of imported topotecan as a second-line chemotherapy agent, yielded an ICER of US$30,076.37/QALY, suggesting the cost-effectiveness of the tislelizumab plus chemotherapy. Generally, these findings highlight significant differences in model selection and imaging assessments between this study and previous ones, reflecting a balanced consideration of cost-effectiveness and practical feasibility. Consequently, our conclusions may carry more weight and practical relevance in economic evaluations.

One-way sensitivity analyses showed that progression-free survival (PFS) had the greatest impact, with an ICER in the range of (0.538–0.808), corresponding to a cost of US$27246–US$ 36,417/QALY, followed by the price of tislelizumab and overall survival (OS). In other similar CEAs, the primary determinant was typically the price of immune checkpoint inhibitors (ICIs). However, in this study, tislelizumab’s price exerted less influence, likely due to its significant cost advantage over alternatives such as atezolizumab, nivolumab, and durvalumab. Since its market launch in December 2019, the price of tislelizumab has undergone several rounds of government-mediated negotiations, decreasing from $1,657/100 mg to $178/100 mg by November 2024, representing an 89% reduction (source: China Data Platform, See text footnote 1). Currently, tislelizumab has not yet been included in the reimbursement list for ES-SCLC. Given the high incidence of ES-SCLC, including tislelizumab in reimbursement schemes may impose a considerable financial burden on the healthcare budget. Cost-effectiveness analysis using the one-time per capita GDP threshold suggests that further price reductions are required for tislelizumab to achieve acceptable cost-effectiveness. Other key drivers identified in this study were consistent with those in similar CEAs, supporting the validity and robustness of the model employed.

There are some limitations to this study. First, the study was mainly based on data from phase III clinical trials and lacked data from real-world studies, so patients of different ages or physical conditions were not adequately considered. Secondly, PD-L1 expression is an important factor influencing the therapeutic effect of immune checkpoint drugs, but since subgroup analysis of PD-L1 expression was not performed in the phase III clinical trial, the present study did not perform PD-L1 subgroup analysis and thus failed to screen the optimal benefit population. In addition, grade 1–2 adverse events were not included. Although grade 1–2 AEs were not included. On the other hand, the result from the univariate analysis indicated that the rate of serious AEs is not a major parameter influencing the ICER, therefore, the omission of grade 1–2 AEs less likely exerted a major impact on the overall results.

## 5 Conclusion

From the perspective of the Chinese healthcare system, using a threshold of three times the per capita GDP, tislelizumab plus chemotherapy is cost-effective compared with chemotherapy alone in the first-line treatment of patients with ES-SCLC. Sensitivity analyses further support this finding, demonstrating that the combination therapy remains highly cost-effective under various assumptions. These results provide robust pharmacoeconomic evidence to support the inclusion of tislelizumab combined with chemotherapy in the reimbursement list as a first-line treatment for ES-SCLC.

## Data Availability

The original contributions presented in the study are included in the article/Supplementary material, further inquiries can be directed to the corresponding author.

## Footnotes

^1^ https://data.yaozh.com/
