# Supplementary material for: Cost-effectiveness analysis of first-line tislelizumab plus chemotherapy for extensive-stage small cell lung cancer from the perspective of the healthcare system in China
Source: Front Public Health. 2025 May 30;13:1552734. doi: 10.3389/fpubh.2025.1552734 (PMC12162963; doi:10.3389/fpubh.2025.1552734)
Supplement: Supplementary file 1 [file Table_1.docx]

Supplementary Material

# Supplementary Data

**Supplementary Table 1** Risk for main AEs and disutility for main AEs

|  | **Baseline value** | **rang** | **Reference** |
| --- | --- | --- | --- |
| **Risk for main AEs in chemo** | **0.79** | **0.632-0.948** | （1） |
| Risk of anemia | 0.17 | 0.136-0.2 | （1） |
| Risk of thrombocytopenia | 0.25 | 0.2-0.3 | （1） |
| Risk of white blood cell decreased | 0.16 | 0.216-0.324 | （1） |
| Risk of decreased neutrophil count | 0.21 | 0.168-0.252 | （1） |
| **Risk for main AEs in Tisle plus chemo** | **0.73** | **0.584-0.876** |  |
| Risk of anemia | 0.16 | 0.128-0.192 | （1） |
| Risk of thrombocytopenia | 0.19 | 0.152-0.228 | （1） |
| Risk of white blood cell decreased | 0.24 | 0.192-0.288 | （1） |
| Risk of decreased neutrophil count | 0.14 | 0.112-0.168 | （1） |
| **Disutility** |  |  |  |
| Anemia | 0.074 | 0.059-0.089 | (2) |
| Decreased neutrophil count | 0.09 | 0.072-0.108 | (2) |
| White blood cell decreased | 0.09 | 0.072-0.108 | (2) |
| Thrombocytopenia | 0.2 | 0.160-0.240 | (3) |
| **Disutility for main AEs in chemo** | **0.142** | **0.113-0.17** |  |
| **Disutility for main AEs in tisleli plus chemo** | **0.126** | **0.11-0.15** |  |

Abbreviations: AEs:adverse events; Chemo:chemotherapy; OS: overall survival; PFS: Progression-Free Survival; Tislel: Tislelizumab

| **Supplementary Table 2 :** comparison of survival models distribution for all populations | | | | |
| --- | --- | --- | --- | --- |
|  | **AIC** | | **BIC** | |
| **PFS** | **Tisleli plus chemo** | **Placebob plus chemo** | **Tisleli plus chemot** | **Placebob plus chemo** |
| **Exponential** | 394.24 | 200.07 | 397.66 | 203.49 |
| **Weibull** | 325.048 | 186.25 | 331.90 | 193.10 |
| **Gamma** | 277.39 | 184.63 | 284.24 | 191.48 |
| **Log-normal** | 245.29 | 194.52 | 252.15 | 201.37 |
| **Gompertz** | 389.85 | 195.18 | 396.70 | 202.03 |
| **Log-logistic** | 210.16 | 181.49 | 217.01 | 188.34 |
| **Gengamma** | 246.83 | 186.33 | 257.11 | 196.60 |
|  | **AIC** | | **BIC** | |
| **OS** | **Tisleli plus chemo** | **Placebob plus chemo** | **Tisleli plus chemo** | **Placebob plus chemo** |
| **Exponential** | 394.24 | 244.05 | 397.66 | 247.48 |
| **Weibull** | 325.05 | 201.49 | 331.90 | 208.34 |
| **Gamma** | 277.39 | 191.76 | 284.24 | 198.61 |
| **Log-normal** | 245.30 | 184.42 | 252.15 | 191.27 |
| **Gompertz** | 389.85 | 227.59 | 396.70 | 234.44 |
| **Log-logistic** | 210.16 | 182.21 | 217.01 | 189.06 |
| **Gengamma** | 246.84 | 186.15 | 257.11 | 196.42 |

Abbreviations: AIC: Akaike Information Criterion ; AEs:adverse events; Chemo:chemotherapy; BIC:Bayesian Information Criterion; OS: overall survival; PFS: Progression-Free Survival; Tislel: Tislelizumab

**Supplementary Table 3:** the results of Scenario analyses 1

| **Group** | **Cost (US$)** | **QALYs** | **Incremental cost (US$)** | **Incremental QALY** | **ICER (US$/QALY)** |
| --- | --- | --- | --- | --- | --- |
| **Tisle+chemo** | 32466.87 | 1.01 | 10515.93 | 0.27 | 38665.59 |
| **Placebo+chemo** | 21950.94 | 0.74 | NA | NA | NA |

Abbreviations: Chemo:chemotherapy; ICER:increment cost-effectiveness ratio; QALY:quality-adjusted life-years. Tislel:Tislelizumab;

**Supplementary Table 4:** The results of Scenario analyses 2

| **Group** | **Cost (US$)** | **QALYs** | **Incremental cost (US$)** | **Incremental QALY** | **ICER (US$/QALY)** |
| --- | --- | --- | --- | --- | --- |
| **Tisle+chemo** | 29537.77 | 1.01 | 8179.91 | 0.27 | 30076.37 |
| **Placebo+chemo** | 21357.86 | 0.74 | NA | NA | NA |

Abbreviations: Chemo:chemotherapy; ICER:increment cost-effectiveness ratio; QALY:quality-adjusted life-years. Tislel: Tislelizumab;

# Reference

1.Cheng Y, Fan Y, Zhao Y, Huang D, Li X, Zhang P, Kang M, Yang N, Zhong D, Wang Z, et al. Tislelizumab Plus Platinum and Etoposide Versus Placebo Plus Platinum and Etoposide as First-Line Treatment for Extensive-Stage SCLC (RATIONALE-312): A Multicenter, Double-Blind, Placebo-Controlled, Randomized, Phase 3 Clinical Trial. Journal of Thoracic Oncology (2024) 19:1073–1085. doi: 10.1016/j.jtho.2024.03.008

2. Zeng X, Li J, Peng L, Wang Y, Tan C, Chen G, Wan X, Lu Q, Yi L. Economic Outcomes of Maintenance Gefitinib for Locally Advanced/Metastatic Non-Small-Cell Lung Cancer with Unknown EGFR Mutations: A Semi-Markov Model Analysis. PLoS ONE (2014) 9:e88881. doi: 10.1371/journal.pone.0088881

3. Zhu Y, Liu K, Wang K, Peng L. Vascular Endothelial Growth Factor Receptor Inhibitors in Chinese Patients With Advanced Radioactive Iodine-Refractory Differentiated Thyroid Cancer: A Network Meta-Analysis and Cost-Effectiveness Analysis. Front Endocrinol (2022) 13:909333. doi: 10.3389/fendo.2022.909333
